# Effect of antiretroviral treatment on blood-brain barrier integrity in HIV-1 infection

**DOI:** 10.1186/s12883-021-02527-8

**Published:** 2021-12-22

**Authors:** Birgitta Anesten, Henrik Zetterberg, Staffan Nilsson, Bruce J. Brew, Dietmar Fuchs, Richard W. Price, Magnus Gisslén, Aylin Yilmaz

**Affiliations:** 1 Department of Infectious Diseases, Institute of Biomedicine, Sahlgrenska Academy, University of Gothenburg, SE-415 50 Gothenburg, Sweden; 2 Department of Infectious Diseases, Region Västra Götaland, Sahlgrenska University Hospital, Gothenburg, Sweden; 3 Department of Psychiatry and Neurochemistry, Institute of Neuroscience and Physiology, Sahlgrenska Academy, University of Gothenburg, Mölndal, Sweden; 4 Clinical Neurochemistry Laboratory Sahlgrenska University Hospital, Mölndal, Sweden; 5 Department of Neurodegenerative Disease, UCL Institute of Neurology, Queen Square, London, UK; 6 UK Dementia Research Institute at UCL, London, UK; 7 Hong Kong Center for Neurodegenerative Disease, Hong Kong, China; 8 Mathematical Sciences, Chalmers University of Technology, Gothenburg, Sweden; 9 Department of Neurology, St.Vincent’s Hospital, Sydney, NSW Australia; 10 Department of HIV Medicine and Peter Duncan Neurosciences Unit, St Vincent’s Centre for Applied Medical Research, St. Vincent’s Hospital, Sydney, NSW Australia; 11 Division of Biological Chemistry, Biocenter, Innsbruck Medical University, Innsbruck, Austria; 12 Department of Neurology, University of California San Francisco, San Francisco, California USA

**Keywords:** HIV, Cerebrospinal fluid, Blood-brain-barrier, Albumin ratio, Antiretroviral therapy, Biomarkers

## Abstract

**Background:**

Blood-brain barrier (BBB) injury is prevalent in patients with HIV-associated dementia (HAD) and is a frequent feature of HIV encephalitis. Signs of BBB damage are also sometimes found in neuroasymptomatic HIV-infected individuals without antiretroviral therapy (ART). The aim of this study was to investigate the integrity of the BBB before and after initiation of ART in both neuroasymptomatic HIV infection and in patients with HAD.

**Methods:**

We determined BBB integrity by measuring cerebrospinal fluid (CSF)/plasma albumin ratios in archived CSF samples prior to and after initiation of ART in longitudinally-followed neuroasymptomatic HIV-1-infected individuals and patients with HAD. We also analyzed HIV RNA in blood and CSF, IgG Index, CSF WBC counts, and CSF concentrations of β2-micoglobulin, neopterin, and neurofilament light chain protein (NfL).

**Results:**

We included 159 HIV-infected participants; 82 neuroasymptomatic individuals and 77 with HAD. All neuroasymptomatic individuals (82/82), and 10/77 individuals with HAD, were longitudinally followed with a median (interquartile range, IQR) follow-up of 758 (230–1752) days for the neuroasymptomatic individuals, and a median (IQR) follow-up of 241 (50–994) days for the individuals with HAD. Twelve percent (10/82) of the neuroasymptomatic individuals and 80% (8/10) of the longitudinally-followed individuals with HAD had elevated albumin ratios at baseline. At the last follow-up, 9% (7/82) of the neuroasymptomatic individuals and 20% (2/10) of the individuals with HAD had elevated albumin ratios. ART significantly decreased albumin ratios in both neuroasymptomatic individuals and in patients with HAD.

**Conclusion:**

These findings indicate that ART improves and possibly normalizes BBB integrity in both neuroasymptomatic HIV-infected individuals and in patients with HAD.

## Background

Human immunodeficiency virus type 1 (HIV-1) invades the central nervous system (CNS) early in primary infection [1]. Over time, in the absence of treatment, this evolves into chronic infection and inflammation that can disrupt the integrity of the blood-brain barrier (BBB). Deterioration of the BBB is most likely multifactorial, with contributing factors including secretion of pro-inflammatory cytokines and viral proteins from infected cells, as well as direct invasion of the cells of the neurovascular unit especially pericytes and perivascular macrophages by HIV [2]. Intrathecal immune activation, as measured by increased levels of neopterin in cerebrospinal fluid (CSF), is associated with impairment of the BBB [3–5]. A non-intact BBB facilitates the influx of proteins, viral particles, and other potentially neurotoxic substances from the blood into the CNS, thereby increasing the risk of cellular injury, including disruption of axons which can be sensitively assessed by measurement of neurofilament light chain protein (NfL) [3, 6]. Several studies have shown an association between BBB integrity, intrathecal immune activation and CSF concentrations of NfL [4, 6–9], implying an association between CNS inflammation, BBB impairment, and neuronal injury.

Prior to the introduction of modern antiretroviral therapy (ART), approximately 30 to 40% of patients with AIDS developed HIV-associated dementia (HAD) [10, 11]. BBB integrity is impaired in patients with HIV encephalitis, and the BBB has been shown to play an important role in the pathogenesis and development of HAD [1, 12–14], although the process has not been fully understood. We have previously determined that 68% of individuals with HAD had signs of BBB impairment [4, 15], but we found no association between the level of immune suppression and elevated albumin ratio in untreated neuroasymptomatic individuals out of whom 16% had signs of BBB impairment [3, 4].

ART drastically reduces CSF HIV RNA levels [16] and CSF concentrations of biomarkers of intrathecal inflammation and neuronal injury [6, 16–18]. However, despite efficient ART with undetectable viral levels in blood and CSF, many individuals have persisting immune activation, both systemically and in the CNS [17–19]. Previous studies have found an altered BBB in up to one-third of untreated neuroasymptomatic individuals [4, 20].

The effect of ART on the BBB has only been studied in a few case reports, where patients with HAD showed normalized BBB function after initiation of ART [21, 22]. The aims of our study were a) to evaluate the effect of ART on BBB integrity over time, b) examine how changes in BBB integrity may be correlated to CNS inflammation and signs of neuronal injury in both neuroasymptomatic HIV-infected individuals and in individuals with HAD, and c) determine whether initiating ART may improve or even normalize BBB function.

## Methods

### Patients

In this retrospective longitudinal study, we analyzed archived CSF samples from HIV-infected adult (≥ 18 years) individuals with no neurological symptoms, and from individuals with HAD, both before and after initiation of ART, which included three active agents: two nucleoside reverse transcriptase inhibitors plus one non-nucleoside reverse transcriptase inhibitors, protease inhibitor, or integrase inhibitor. The samples were collected from 23 September 1986 until 1 June 2017 at three locations: Sahlgrenska University Hospital, Gothenburg, Sweden; San Francisco General Hospital, San Francisco, CA, USA, and St. Vincent’s Hospital, Sydney, Australia. To meet the inclusion criteria, participants showing no neurological symptoms had to be treatment-naïve, or off treatment for a minimum of 6 months prior to initiation of ART. To study the effect of efficient ART, they had to reach viral suppression within 7 months and remain virally suppressed for more than 6 months. Viral suppression within 7 months was chosen to allow individuals who had a reduction in plasma HIV RNA levels as expected on treatment, but where the lumbar puncture was not performed exactly at 6 months. Blips with a maximum of 500 HIV RNA copies/mL at the time of lumbar puncture during follow-up were considered acceptable. For individuals who had > 1 valid sample date according to the inclusion criteria, the most recent sample before initiation of ART was included in the study. Neuroasymptomatic participants were clinically assessed for neurocognitive and neurologic symptoms. Those who had neurologic symptoms or complaints at baseline, or developed during follow up, were excluded in order to more clearly evaluate the BBB and the associations with other biomarkers. No routine neurocognitive testing was performed on neuroasymptomatic patients.

Participants without neurologic signs or complaints were designated neuroasymptomatic. The diagnosis of HAD was based on the Centers for Disease Control and Prevention and American Academy of Neurology Task Force criteria, using standard laboratory, neuropsychological, and clinical evaluations [23, 24]. Treatment was initiated within 3 months of pre-ART samples in the case of all participants. Individuals with primary HIV infection, CNS opportunistic or other infections, CNS neoplasms, acute cranial nerve paresis, or neurologic diseases with CNS involvement were excluded.

Inclusion in the HAD cohort required a lumbar puncture sample both prior to and while on ART. Participants had to be treatment-naïve or off treatment for 6 months or more, and must have reached viral suppression. In accordance with the inclusion criteria participants with HAD who recovered on treatment were not excluded. Since HAD is a rare condition and few individuals had been followed longitudinally prior to and during ART, we also included individuals with HAD who had a lumbar puncture either prior to initiation of ART or while on ART, and who had been virally suppressed for 6 months or more.

Lumbar punctures during follow up in all participants were performed according to different schedules depending on the research site. In Gothenburg, lumbar punctures after initiation of ART were made after 3 months, 12 months, and yearly thereafter. In San Francisco lumbar punctures were made frequently during the first 3 months and thereafter with a few months to 1 year’s interval. From Sydney, only cross-sectional data on patients with or without ART was provided.

CSF samples were obtained in accordance with research protocols approved by the institutional review boards of each of the study sites, and in compliance with the Helsinki Declaration, either within the context of studies of the natural history of HIV infection (for neuroasymptomatic individuals and those on ART), or during diagnostic evaluations (for patients with HAD). All participants gave their informed consent or, if their capacity to provide consent was questioned, consent was also obtained from those with power of attorney.

### Methods

Quantitative determinations of CSF and plasma albumin was performed with nephelometry (Behring Nephelometer Analyser, Behringwerke AG, Marburg, Germany). Albumin ratio was calculated as CSF albumin (mg/L)/plasma albumin (g/L) and used to evaluate BBB function. Reference values were < 6.8 for individuals younger than 45 years and <  10.2 for individuals ≥45 years of age [15].

CSF neopterin was analyzed using a commercially available immunoassay (BRAHMS, Berlin, Germany) according to the manufacturer’s instructions. The normal upper reference value for CSF neopterin is 5.8 nmol/L. [25] CSF NfL concentration was measured by a sandwich enzyme-linked immunosorbent assay (NF-light ELISA kit, UmanDiagnostics AB, Umeå, Sweden), with age adjusted values. Mean adjusted age was 44 years and upper normal reference value was 823 ng/L. [26] HIV RNA levels in blood and CSF were quantified by the Roche Amplicor Monitor version 1.5 (Roche Molecular Systems, Branenburg, NJ) Abbott RealTime HIV-1 assay (Abbott Laboratories, Abbott Park, IL), or Roche Taqman assay, version 1 or 2 (Hoffman La-Roche, Basel, Switzerland). The lower limit of quantification has changed over the years due to the implementation of new PCR methods, and varied between 20 and 200 HIV RNA copies/mL, depending on the different assays. Viral suppression was defined as a plasma HIV RNA level below the limit of detection, measured with the method used at the time. CSF β2-microglobulin was measured using the N Latex β2M kit on the Atellica NEPH 630 System (Siemens Healthcare GmbH. Erlangen, Germany). Blood CD4^+^ T-cell counts, CSF leukocytes (WBC), IgG index, and CSF/blood albumin measurements were performed at the local clinical laboratories. IgG index as a marker of intrathecal IgG synthesis was defined as [CSF IgG (mg/L)/serum IgG (g/L)]/[CSF albumin (mg/L)/serum albumin (g/L)] [27]. The reference value for IgG Index was < 0.63, independent of age [15]; and for CSF β2-microglobulin it was < 1.2 mg/L for individuals 18–49 years and <  1.8 mg/L for individuals ≥50 years of age.

### Statistical analysis

Descriptive statistics were performed using Prism, version 9.0 (GraphPad, La Jolla, CA) or SPSS, version 27 (IBM, Armonk, NY) or R, version 4.0, software. All continuous variables except age and leukocyte count were log_10_ transformed to reduce skewness.

Associations were analyzed with Pearson correlation coefficients. Independent Student’s t-test was used for comparison between two groups, and Wilcoxon signed rank test was used for evaluation over time at 6 months and 3 years with interpolated values using LOCF (last observation carried forward) if all values were prior to these time points.

## Results

In total, we included 159 HIV-infected participants; 82 neuroasymptomatic individuals, and 77 with HAD. All of the neuroasymptomatic individuals and 10 of those with HAD were followed longitudinally with lumbar punctures both pretreatment and during ART. Sixty-seven of the participants with HAD had lumbar punctures either only pre-ART (*n* = 47) or on ART (*n* = 20); for these individuals only cross-sectional data were available (Table 1). Median (interquartile range [IQR]) follow-up was 25 months (8–59) for neuroasymptomatic individuals, with a maximum follow-up for those on ART of 15 years. For individuals with HAD, median follow-up was 8 (2–33) months, with a maximum follow-up on ART of 11 years. Untreated individuals with HAD (cross-sectionally and longitudinally-followed) did not have significantly higher plasma HIV RNA, but significantly higher CSF HIV RNA (*p* < 0.0001), albumin ratio (*p* < 0.0001), CSF neopterin (*p* < 0.0001), and CSF NfL (*p* < 0.0001), and significantly lower CD4^+^ T-cell count (*p* < 0.0001) and CD4 nadir (*p* < 0.0001), compared to neuroasymptomatic participants. There was no significant difference in age between the groups.

**Table 1:** Baseline patient characteristics

	Longitudinal		Cross-sectional	
Characteristic	NA	HAD	HAD untreated	HAD treated
	(***n*** = 82)	(***n*** = 10)	(n = 47)	(n = 20)
Gender: male, number (%)	49 (60)	9 (90)	44 (94)	18 (90)
Age (years)	38 (31–46)	41 (35–46)	40 (34–45)	45 (38–52)
CD4+ T-cell count (× 106 cells/L)	210 (70–330)	68 (60–137)	90 (17–175)	49 (38–52)
CD4+ nadir T-cell count (× 106 cells/L)	180 (60–250)	52 (16–80)	141 (18–160)c	65 (50–130)e
Plasma HIV RNA (log10 copies/mL)	4.79 (4.32–5.42)	5.49 (5.32–5.78)	4.87 (1.72–5.50)	< 1.70
CSF HIV RNA (log10 copies/mL)	3.78 (3.23–4.38)	5.23 (5.09–5.41)	4.16 (2.78–4.98)	< 1.70
Albumin ratio	4.26 (3.57–6.00)	10.6 (7.50–17.24)	9.90 (5.72–13.23)	6.57 (4.87–7.36)
CSF neopterin (nmol/L)	18.1 (11.5–24.0)	50.4 (43.1–85.8)a	50.0 (27.2–95.5)	13.5 (9.6–17.9)
CSF NfL (ng/L)	422 (318–713)	14,115 (9824–23,543)b	9731 (7603–21,555)d	f
Follow-up, days	758 (230–1752)	241 (50–994)	N/A	N/A

If not stated otherwise, values are presented as median (IQR). *IQR* interquartile range, *CSF* cerebrospinal fluid, *NA* neuroasymptomatic, HAD: HIV-associated dementia. Missing values: a2/10, b4/10, c40/47 d43/47, e15/20, fno values. CSF NfL as age-adjusted values

### Neuroasymptomatic individuals

Among the longitudinally-followed neuroasymptomatic individuals, 12% (10/82) had an elevated albumin ratio at baseline. Baseline characteristics for these ten did not differ compared to the others in the neuroasymptomatic cohort. At the last follow-up 9 % (7/82) had elevated albumin ratios.

**Fig. 1:**
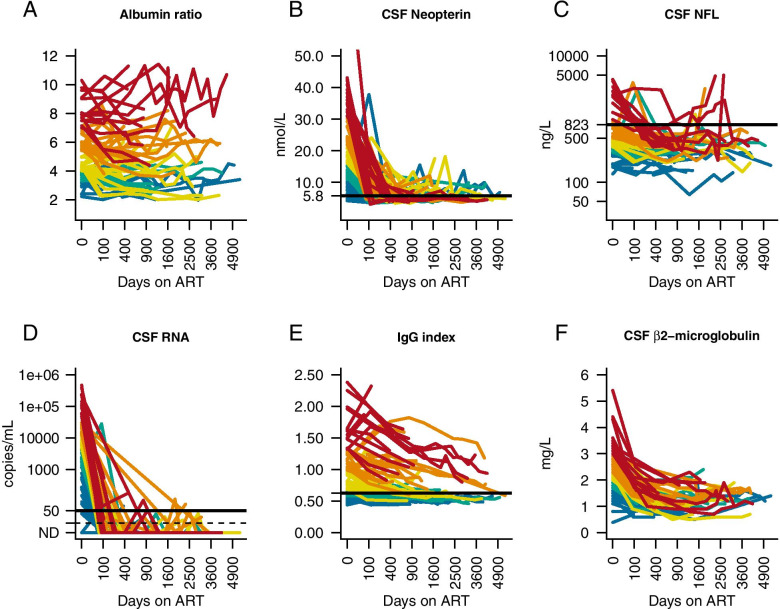
Changes in biomarkers after initiation of antiretroviral therapy (ART) in neuroasymptomatic HIV-infected individuals. (**A**) albumin ratio, (**B**) CSF neopterin, (**C**) age-adjusted CSF NfL, (**D**) CSF HIV RNA, (**E**) IgG index, (**F**) CSF β2-microglobulin. Colors represent levels of albumin ratio at baseline by quantiles (red indicates the highest quantile, orange the second highest, yellow the third highest, green the fourth highest and blue the quantile with the lowest baseline levels). Horizontal black lines represent upper normal reference value, except (**D**), which shows 50 viral copies/mL and dotted black line, which shows 20 viral copies/mL. Scale of X-axis is square root of days since ART initiation

After initiation of ART, the albumin ratio decreased significantly as measured at two defined time points: 6 months (*p* < 0.05) and 3 years (*p* < 0.01) (Fig. 1). At the same time points, CSF HIV RNA (*p* < 0.001, *p* < 0.001), CSF neopterin (*p* < 0.001, *p* < 0.001), CSF β2-microglobulin (*p* < 0.001, *p* < 0.001), WBC (*p* < 0.001, *p* < 0.001), and CSF NfL (*p* < 0.01, *p* < 0.001) also decreased significantly. There was a trend towards a significant decrease in the IgG index at 6 months (*p* = 0.06), and a significant decrease after 3 years of treatment (*p* < 0.001) (Fig. 1).

At baseline, there was an inverse correlation between albumin ratio and IgG Index (r = − 0.4, *p* < 0.001) and a significant correlation with age (r = 0.4, *p* = 0.001). No correlations were found between albumin ratio and CSF NfL, inflammatory markers such as CSF neopterin, β2-microglobulin, or WBC; nor with plasma or CSF HIV RNA, CD4^+^ T-cell count, or CD4 nadir.

### Individuals with HAD

**Fig. 2:**
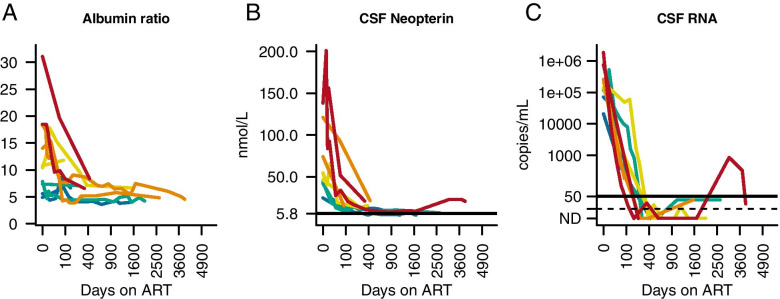
Changes in biomarkers after initiation of antiretroviral therapy (ART) in individuals with HIV-associated dementia (HAD). (**A**) albumin ratio, (**B**) CSF neopterin, (**C**) CSF HIV RNA. Colors represent levels of albumin ratio at baseline by quantiles (red indicates the highest quantile, orange the second highest, yellow the third highest, green the fourth highest and blue the quantile with the lowest baseline levels). Horizontal black lines represent upper normal reference value, except (**C**), which shows limit of detection of viral copies/mL. (CSF β2-microglobulin, IgG index, and CSF NfL not analyzed due to lack of samples.) Scale of X-axis is square root of days since ART initiation

Eight out of the ten longitudinally-followed individuals with HAD had an elevated albumin ratio at baseline. Among those untreated individuals with HAD who had only one cross-sectional lumbar puncture, 68% (32/47) showed elevated albumin ratios. In the cross-sectional HAD cohort of those with only one lumbar puncture who were on ART, 25% (5/20) had elevated albumin ratios. In the longitudinal HAD cohort, 20% (2/10) had elevated albumin ratios at their last follow-up. For these ten individuals with HAD who were longitudinally followed, the albumin ratio declined, although not significantly, at 6 months; and significantly after 3 years (*p* < 0.05). At the same defined time points, there was a significant reduction of CSF HIV RNA (*p* < 0.01, *p* < 0.01) and CSF neopterin (*p* < 0.01), *p* < 0.01), (Fig. 2). WBC, CSF β2-microglobulin, IgG index, and CSF NfL were not analyzed due to lack of data.

In the 57 untreated individuals with HAD (cross-sectionally and longitudinally-followed), no correlation was found between albumin ratio and plasma HIV RNA, CSF HIV RNA, CD4^+^ T-cells, CD4 nadir, WBC, IgG Index, CSF neopterin, or CSF NfL, but a significant correlation was found with CSF β2-microglobulin (r = 0.7, *p* < 0.05).

**Fig. 3:**
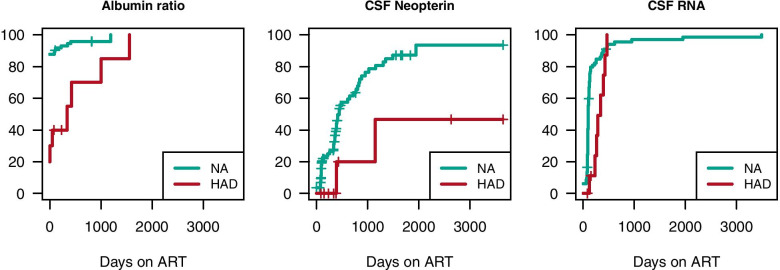
Time to normalization of albumin ratio, CSF neopterin, and CSF HIV RNA. Many neuroasymptomatic individuals (NA), in green lines, showed early reduction of CSF HIV RNA and CSF neopterin. Reduction of CSF HIV RNA, CSF neopterin, and albumin ratio occurred later in individuals with HIV-associated dementia (HAD), in red lines. The plateau of CSF neopterin was higher in the HAD group. (CSF β2-microglobulin, IgG index, and CSF NfL not analyzed due to lack of samples)

In all longitudinally followed individuals (82 neuroasymptomatic and 10 with HAD), the decrease in CSF viral load and neopterin occurred early after initiation of ART, whereas the decrease in CSF β2-microglobulin and IgG index occurred later. Despite the normalization of CSF HIV RNA, levels of inflammatory markers in CSF remained slightly elevated. In individuals with HAD, the plateau of elevated CSF neopterin was higher than for neuroasymptomatic individuals (Fig. 3).

## Discussion

In our study we found that BBB integrity improved after initiation of ART in both neuroasymptomatic individuals and in those with HAD. A small proportion (12%) of neuroasymptomatic individuals had elevated albumin ratios before initiation of ART, as opposed to 80% of the individuals with HAD. This is in the same range as in one of our previous cross-sectional studies (*n* = 631), where 16% of untreated neuroasymptomatic individuals showed elevated albumin ratios. It also is in accordance with other studies [3, 4, 12, 13, 28]. However, in an Italian study with 44 ART-naïve participants, 39% had elevated albumin ratios. One explanation for the discrepancy could be that in our two studies, lumbar punctures were performed in a research setting, whereas in the Italian study they were done for clinical purposes, such as the investigation of neurocognitive disorders; for differential diagnosis, such as epilepsy, hepatic, or vascular encephalopathy; or for sudden onset of psychiatric disorders when BBB disturbances could be part of other, non-HIV specific, morbidities. Also, one-third of the participants reported intravenous drug abuse [4, 20]. The latter can affect the integrity of the BBB [29–31]. In our present study, only four individuals, all with HAD, had a history of drug abuse, and one reported current drug use. In another recent Italian study, 35% of 147 ART-naïve individuals showed BBB impairment. However, only 6% of the participants were neuroasymptomatic [32]. The remaining individuals were diagnosed with a variety of CNS disorders, including neurosyphilis, opportunistic infections, and HIV-associated neurocognitive disorders (HAND), making it difficult to draw comparisons with our studies.

Since the majority of the neuroasymptomatic individuals we examined had albumin ratios within the normal range, it is not surprising that the ratios remained fairly stable over time after initiation of ART. The neuroasymptomatic individuals whose BBB was impaired at baseline were similar in immune status and other baseline data. There was a slight, although not significant, difference in age: a median age of 40 years in those with elevated albumin ratios compared to 38 years for those with a normal albumin ratio, which would not explain the discrepancy between baseline levels of albumin ratios. The duration of HIV disease could not be assessed, this may be a cause for the discrepancy. Another possible explanation may be that the BBB impairment is not related to the HIV-infection. We have previously found that neuroasymptomatic HIV-infected individuals have albumin ratios in the same range in as those of HIV negative controls [4], which indicates that there could be other factors associated with BBB impairment, such as drug abuse (i.e., methamphetamine and cocaine) [29, 30], and environmental factors [33]. In a recent study, HIV-negative individuals on pre-exposure prophylaxis (PrEP) had significantly higher albumin ratios compared with healthy HIV-negative individuals not on PrEP, reflecting a similarity in life-style related factors to people living with HIV [34].

In agreement with the findings of earlier research, the neuroasymptomatic individuals in our study had a significant reduction of CSF viral load [35, 36] and inflammatory markers such as CSF neopterin and β2-microglobulin after initiation of ART, including a rapid decline of CSF neopterin, also seen in previous studies [17, 37]. However, the latter markers did not completely normalize, indicating an ongoing intrathecal immune activation, despite viral control in both plasma and CSF [17, 19]. We found no correlation between albumin ratio and CSF neopterin, in contrast to previous findings [3, 4]. A possible explanation could be that baseline albumin ratios were slightly lower in this cohort than in the larger study we conducted earlier (4.26 vs 4.90 in median), or that it was due to the different sample sizes. Age did not differ between the cohorts (median 38 years in both groups).

The albumin ratio in individuals with HAD also declined, but was not statistically significant until the second time point, at 3 years, indicating that improvement of the BBB takes longer in individuals with HAD. Viral replication in individuals with HAD primarily takes place in cells in brain tissue or in tissue macrophages rather than in CSF, as for neuroasymptomatic individuals. This may be reflected in the slower reduction of HIV RNA after ART initiation. Nevertheless, a majority (75%) of those with elevated albumin ratios at baseline normalized this following the initiation of ART.

An intact BBB is vital to maintain homeostasis in the CNS and protect the brain from peripheral, potentially neurotoxic, substances. It is composed of a semipermeable layer of a highly specialized capillary bed that surrounds the CNS. The endothelial cells are fused together with tight junctions [38]. Viral proteins such as Tat, Gp120, and Nef are produced when HIV infects cells in the periphery and in the CNS. Together with inflammatory mediators (e.g., cytokines and chemokines), they can damage the BBB by impacting less tight junctions and facilitating permeability [39, 40]. This enables an increased influx of blood and viral proteins, and other potentially toxic molecules into the CNS. We have previously found an association between BBB damage and axonal injury in neuroasymptomatic individuals (*n* = 415) [4]. The role of the BBB in the progression to HAD (or to milder cognitive impairment) is not yet fully understood but previous research has suggested an association between continuous CNS inflammation, neuronal injury, and reduced BBB integrity [2, 3, 6]. An impaired BBB is predominantly seen in HIV-infected individuals with advanced disease such as HAD, but can also occur in neuroasymptomatic patients, whether they are treatment-naïve or on effective ART [4]. We have previously found that untreated neuroasymptomatic individuals with varying CD4+ T-cell counts have albumin ratios in the same range. This may indicate that HIV-related deterioration of the BBB is a late and perhaps abrupt event. In this study, most neuroasymptomatic individuals had an albumin ratio in the normal range, but with a low CD4+ nadir indicating rather advanced disease, emphasizing that BBB disruption occurs late during the disease course. Due to few neuroasymptomatic individuals with CD4+ T-cell count ≥500, these individuals could not be analyzed separately, but in our previously mentioned study we found that of 125 untreated neuroasymptomatic individuals with CD4+ T-cell count ≥350, 11% had an elevated albumin ratio [4].

CSF/plasma albumin ratio is a reliable marker of BBB impairment [15, 27], and is the most commonly used method to describe such impairment. Other markers have been linked to BBB integrity, including CSF levels of the soluble urokinase plasminogen activator receptor (suPAR) [41–45] and levels of CSF matrix metalloproteinase-3 (MMP-3) [34]. However, to date there is no convincing evidence that CSF levels of SuPAR or CSF MMP-3 offer any additional information on BBB integrity than albumin ratios.

Our study has several limitations. First, the sample size was fairly small, especially for the HAD cohort. As HAD is rare nowadays it is difficult to include many such individuals, although, to the best of our knowledge, ours is at present the largest study. Second, the number of samples during follow-up differs considerably between individuals, some having been followed for many years, and others only a few months. Also, some individuals had a very short interval before their first sample was taken, whereas others had been on ART for several years before their first follow-up sample. Due to a lack of some data in individuals with HAD, important biomarkers, such as IgG index, CSF β2-microglubulin, and CSF NfL, could not be analyzed during follow-up. An additional consequence of the currently rare HAD diagnosis is that data had to be collected over a very long period of time, during which ART has continued to be developed, something which also might have influenced results.

## Conclusions

In conclusion, albumin ratios declined significantly after initiation of ART in neuroasymptomatic HIV-infected individuals, as well as in patients with HAD, although the decline took longer for the latter group, indicating that healing may be slower in individuals with a more pronounced BBB injury. This would confirm the central role of BBB in the process of neuroinflammation and neuronal injury.

## Data Availability

The datasets used and/or analyzed during the current study are available from the corresponding author on reasonable request.

## Abbreviations

ART: Antiretroviral therapy
BBB: Blood-brain barrier
CNS: Central nervous system
CSF: Cerebrospinal fluid
HAD: HIV-associated dementia
HAND: HIV-associated neurocognitive disorder
HIV-1: Human immunodeficiency virus type 1
IQR: Interquartile range
LOCF: Last observation carried forward
MMP-3: Matrix metalloproteinase-3
NfL: Neurofilament light chain protein
PrEP: Pre-exposure prophylaxis
SuPAR: Soluble urokinase plasminogen activator receptor
