# Correction: Effect of antiretroviral treatment on blood-brain barrier integrity in HIV-1 infection

**DOI:** 10.1186/s12883-022-02725-y

**Published:** 2022-05-27

**Authors:** Birgitta Anesten, Henrik Zetterberg, Staffan Nilsson, Bruce J. Brew, Dietmar Fuchs, Richard W. Price, Magnus Gisslen, Aylin Yilmaz

**Affiliations:** 1 Department of Infectious Diseases, Institute of Biomedicine, Sahlgrenska Academy, University of Gothenburg, SE-415 50 Gothenburg, Sweden; 2 Department of Infectious Diseases, Region Vastra Gotaland, Sahlgrenska University Hospital, Gothenburg, Sweden; 3 Department of Psychiatry and Neurochemistry, Institute of Neuroscience and Physiology, Sahlgrenska Academy, University of Gothenburg, Molndal, Sweden; 4 Clinical Neurochemistry Laboratory Sahlgrenska University Hospital, Molndal, Sweden; 5 Department of Neurodegenerative Disease, UCL Institute of Neurology, Queen Square, London, UK; 6 UK Dementia Research Institute at UCL, London, UK; 7 Hong Kong Center for Neurodegenerative Disease, Hong Kong, China; 8 Mathematical Sciences, Chalmers University of Technology, Gothenburg, Sweden; 9 Department of Neurology, St.Vincent’s Hospital, Sydney, NSW Australia; 10 Department of HIV Medicine and Peter Duncan Neurosciences Unit, St Vincent’s Centre for Applied Medical Research, St. Vincent’s Hospital, Sydney, NSW Australia; 11 Division of Biological Chemistry, Biocenter, Innsbruck Medical University, Innsbruck, Austria; 12 Department of Neurology, University of California San Francisco, San Francisco, California USA

**Correction: BMC Neurol 21, 494 (2021)**

**https://doi.org/10.1186/s12883-021-02527-8**

Following publication of the original article [1], an error was reported in the reference list wherein reference 20 has an incorrect journal name.

Incorrect:

- Calcagno A, Alberione MC, Romito A, Imperiale D, Ghisetti V, Audagnotto S, et al. Prevalence and predictors of blood-brain barrier damage in the HAART era. **J Neuro-Oncol**. 2014;20:521–5.

Correct:

- Calcagno A, Alberione MC, Romito A, Imperiale D, Ghisetti V, Audagnotto S, et al. Prevalence and predictors of blood-brain barrier damage in the HAART era. **J Neurovirol**. 2014;20:521-5.

The original article [1] has been updated.
